# Assessing the reporting of categorised quantitative variables in observational epidemiological studies

**DOI:** 10.1186/s12913-017-2137-z

**Published:** 2017-03-14

**Authors:** Onkabetse V. Mabikwa, Darren C. Greenwood, Paul D. Baxter, Sarah J. Fleming

**Affiliations:** 1 Division of Epidemiology and Biostatistics, LICAMM, School of Medicine, University of Leeds, Leeds, UK; 2 Section of Epidemiology and Biostatistics, LICAP, School of Medicine, University of Leeds, Leeds, UK

**Keywords:** Categorisation, Quantitative or continuous variables, STOBE guidelines, Observational studies

## Abstract

**Background:**

One aspect to consider when reporting results of observational studies in epidemiology is how quantitative risk factors are analysed. The STROBE (Strengthening the Reporting of Observational Studies in Epidemiology) guidelines recommend that researchers describe how they handle quantitative variables when analysing data. For categorised quantitative variables, the authors are required to provide reasons and justifications informing their practice. We investigated and assessed the practices and reporting of categorised quantitative variables in epidemiology.

**Methods:**

The assessment was based on five medical journals that publish epidemiological research. Observational studies published between April and June 2015 and investigating the relationships between quantitative exposures (or risk factors) and the outcomes were considered for assessment. A standard form was used to collect the data, and the reporting patterns amongst eligible studies were quantified and described.

**Results:**

Out of 61 articles assessed for eligibility, 23 observational studies were included in the assessment. Categorisation of quantitative exposures occurred in 61% of these studies and reasons informing the practice were rarely provided. Only one article explained the choice of categorisation in the analysis. Transformation of quantitative exposures into four or five groups was common and dominant amongst studies using equally spaced categories. Dichotomisation was not popular; the practice featured in one article. Overall, the majority (86%) of the studies preferred ordered or arbitrary group categories. Other criterions used to decide categorical boundaries were based on established guidelines such as consensus statements and WHO standards.

**Conclusion:**

Categorisation of continuous variables remains a dominant practice in epidemiological studies. The reasons informing the practice of categorisation within published work are limited and remain unknown in most articles. The existing STROBE guidelines could provide stronger recommendations on reporting quantitative risk factors in epidemiology.

**Electronic supplementary material:**

The online version of this article (doi:10.1186/s12913-017-2137-z) contains supplementary material, which is available to authorized users.

## Background

Most studies in medicine exhibit serious weaknesses due to issues of reporting [1, 2]. Inadequate and poor reporting practices restrict generalisability and implementation of results and subsequently the clinical and scientific utility of such studies is lost [2–4]. To aid reporting in epidemiology, the STROBE (Strengthening the Reporting of Observational Studies in Epidemiology) [4] and STRATOS (Strengthening Analytical Thinking for Observational Studies) [1] guidelines were developed to guide researchers working on observational studies.

Realising the benefits of research might be achieved slowly without sufficient clarity on reporting; in 2004, researchers, methodologist and journal editors met in a 2-day workshop under the STROBE initiative and developed recommendations (checklist of 22 items) necessary for an accurate and complete observational study [4]. The established recommendations aim at contributing to the improvement of reporting in three main study designs of analytical epidemiology: cohort, case-control designs and cross-sectional studies [4]. One aspect to consider when presenting results of observational studies in epidemiology is how quantitative or continuous risk factors are analysed and reported. The STROBE guidelines recommend authors describe how they handle quantitative variables when analysing the data; for categorised quantitative variables, the guidelines require researchers to explain and justify the methods of categorisation. However, reviews in 2004 and 2010 suggest that few studies at that time were reporting the issues of categorisation in epidemiology appropriately [5, 6]. These suggested that most continuous variables were categorised for analysis and presentation and that the basis for categorisation was rarely described. To investigate whether the analysis and presentation have improved in this area in the past 6 years, we aimed to assess the practice of categorisation in the field of epidemiology.

Categorisation is defined as the practice of converting quantitative or continuous exposures or risk factors such as age, body mass index (BMI) and blood pressure (BP) into two or more groups by splitting them at some points and designating individuals above or below the points as separate groups [7]. For example, age could be divided into several age groups such as 1–5, 6–10, and 10+ or below/above 25^th^, 50^th^ or 75^th^ percentiles or based on quantiles (e.g., tertiles, quartiles, quintiles or deciles). Exposure or risk factors assuming any two distinct values such as gender (coded 0 or 1 for male or females respectively) and medication use (coded 0 or 1 for No and Yes respectively) are known as binary variables. Consequences of categorisation include possible loss of information and statistical power [8], efficiency [9], reliability [7] and higher type I [10] and type II [11] errors, leading to potential misleading estimates and clinical interpretations [8, 12–20].

This study highlights key issues necessary for improvement when reporting and analysing continuous variables in observational studies. The results have relevance to authors and readers working with observational studies in epidemiology. Improved reporting is necessary to promote and preserve scientific knowledge for synthesis and clinical decision making.

## Methods

We based our assessment on five journals we would anticipate to be examples of current best practice in clinical epidemiology, using the highest impact factor (IF) ratings from the Web of Science citation report of July 2015 [21]. Three journals were selected in the area of epidemiology and two general medical journals that publish epidemiological research. Journals selected were the International Journal of Epidemiology, Epidemiology, Journal of Clinical Epidemiology, the New England Journal of Medicine and Lancet. The rationale behind the selection of the five journals was based on impact factor to include journals with high levels of influence in the literature. The common use of categorisation in these leading journals would suggest the method is also widely applied in other journals with lower impact factors or more in specialist journals.

### Study selection

For eligible articles, we considered observational studies published between 1^st^ April and 30^th^ June 2015. Articles published between this time intervals were selected to reflect current practice. Consideration was given to all publications with at least one independent continuous variable in the analysis. Specific eligibility criteria are as follows:

i. Publications based on individual’s data quantifying the risk or association between quantitative exposures and outcomes.
ii. The reported data should be from the original study. The study should not report pooled estimates in the form of systematic reviews and meta-analysis
iii. The study should be based on observational designs such as cohort, case-control and cross-sectional (a requirement in the STROBE guidelines).

#### Exclusion criteria

**Fig. 1:**
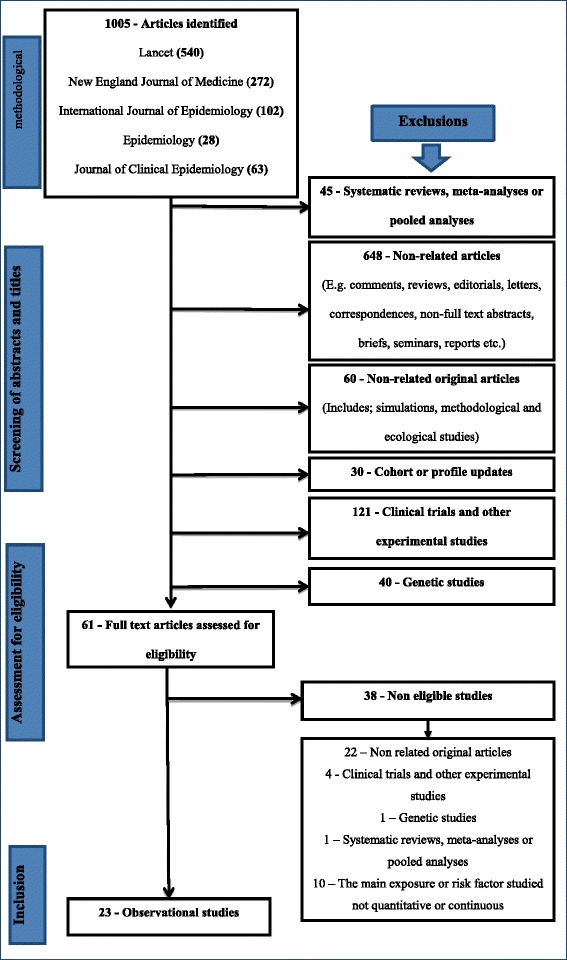
A detailed flow chart summarising the selection and identification process of eligible articles

We excluded all systematic reviews or meta-analyses, clinical trials or experimental studies and genetic epidemiology studies. Epidemiological studies other than cohort, cross-sectional and case-control studies such as ecological studies were also excluded because they are not covered by the STROBE recommendations. Additionally, non-related articles (e.g., comments, correspondence, editorials, non-full text abstracts) and non-related original (full text) publications (e.g., simulations, methodological papers) were also excluded. Details are provided in Fig. 1.

#### Search strategy

The search for eligible articles was done amongst all publications obtained in the five journals. We reviewed all publications to identify those investigating associations between risk factors and disease outcomes or any measures in individuals. The search was done electronically, and the identified articles were later reviewed in more detail. Figure 1 presents a summary of the identification and selection process for eligible articles.

As shown in Fig. 1, we identified 1005 articles from the five Journals: Lancet (540), NEJM (272), IJE (102), Epidemiology (28) and Journal of Clinical Epidemiology (63). From the 1005 publications identified, 944 articles were excluded after screening through their abstracts and titles. Reasons for excluding an article’s title or abstract were based on studies identified and classified as follows; systematic reviews, meta-analyses or pooled analyses (45), non-related articles (648), non-related original articles (60) and cohort or profile update studies (30), clinical trials and other experimental studies (121) and genetic studies (40).

The screening resulted in 61 articles which were retrieved and reviewed as full-text for inclusion in the analysis; 23 observational studies met the eligibility criteria, and 38 were excluded (see Fig. 1). Amongst the 38 studies which were excluded, 22 were not related to the objective of the review, four were clinical trials and other experimental studies, two were meta-analyses and genetic studies and the other ten studies investigated exposures or risk factors which were not quantitative or continuous.

### Data extraction

We used a modified data collection form prepared by Turner et al. [6] in their previous survey (see Additional file 1). The study variables and characteristics collected through this form are as follows: title of the study, lead author surname, date of publication, journal name, type of study design, sample size or number of participants, outcomes and exposures or risk factor characteristics (e.g., specialty, types, and whether they are categorised), details of grouping or categorisation, details of other adjusted variables included in the study, presentation and types of statistical results used in reporting, type of effect estimates (e.g., odds ratios, relative risks, confidence intervals, *p*-values).

### Statistical analysis

The data collected was captured in a Microsoft Access database and exported to Stata 13 for analysis [22]. The patterns of reporting for observational studies were quantified and reported using proportions. Where possible, examples from the data are provided for illustration. Only predominant findings or issues and practices of categorisation are reported.

## Results

### General characteristics

In this section, we provide a summary of results describing general characteristics of 23 observational studies included in the study. Overall, the three epidemiological journals produced 57% (CI = 34%, 77%) of total articles included in the study. The other articles - 43% (CI = 23%, 66%) were obtained from the New England Journal of Medicine and Lancet. The International Journal of Epidemiology (IJE) and Lancet contributed more articles in the study than the other journals. The IJE contributed 39% (CI = 20%, 61%) of the total articles whilst from the Lancet we obtained 35% (CI = 16%, 57%) of the total articles. Amongst these articles, cohort or follow-up studies were common. We obtained 74% (CI = 52%, 90%) of cohort or follow-up studies. The other study designs included; cross-sectional and case-control with 17% (CI = 5%, 39%) and 9% (CI = 1%, 28%) respectively.

Non-communicable diseases such as diabetes, cancer, heart diseases and mental illness were commonly studied contributing 35% (CI = 16%, 57%) amongst principal diseases or outcomes being investigated and mortality followed with 30% (CI = 13%, 53%). HIV, physiological or biochemical markers such as anti-mullerian hormone (AMH) concentration levels, body mass index (BMI) and other conditions contributed 35% (CI = 16%, 57%). These outcome variables were commonly analysed as binary variables (44%, CI = 23%, 66%), continuous variables (30%, CI = 13%, 53%) and time-to-event variables (26%, CI = 10%, 48%). For binary and time-to-event studies, mortality was more predominant compared to other outcome variables.

Considering the exposures or main risk factor variables, socioeconomic exposures were commonly investigated; 30% (CI = 13%, 53%) of studies with such exposures were obtained. For example, Zhang and colleagues [23] investigated the associations between neighborhood deprivation index (socioeconomic exposure) and BMI (outcome). The neighborhood deprivation index in this study was derived from the 2000 US Census housing and population data using variables such as income, poverty, housing, education, and employment and occupation status. The other exposures found included; diet and lifestyle exposures (17%, CI = 5%, 39%), environmental exposures (13%, CI = 3%, 34%) physiological or biochemical markers (9%, CI = 1%, 28%) pre-existing conditions (4%, CI = 0%, 22%) and other varied risk factors (26%, CI = 10%, 48%).

### Incidence of categorisation amongst the exposures or main risk factors

Amongst the 23 studies, 61% (CI = 39%, 80%) transformed the continuous exposures or the main risk factor variables into categorical or grouped measures for analysis. The other 39% (CI = 20%, 61%) kept the exposures or the main risk factor variables continuous. For example, Li and colleagues [24] investigated the association between BMI trajectories and adult BP across two generations keeping the exposure (BMI) continuous. Linear spline function with one knot was used to summarise longitudinal changes of the BMI curves in the two generations. In another example, Victora and colleagues [25] investigated the association between intelligence quotient (IQ) and breastfeeding duration (measured in months) and categorised the exposure (breastfeeding duration). The assumed categories for the exposure were varied, defined according to the total duration of breastfeeding and predominant breastfeeding duration (breastfeeding as the main form of nutrition with some other foods). The total duration of breastfeeding (in months) was categorised using five interval groups; <1, 1–2.9, 3–5.9, 6–11.9 and ≥ 12 which differed to the predominant breastfeeding categories defined as; <1, 1–1.9, 2–2.9, 3–3.9 and ≥4. In most articles, whenever categorical analysis was deployed as in the latter example, the categories were assigned ordinal values or scores to depict distinct levels amongst the categorised groups. Further details on the practices of categorisation considering only articles where continuous exposures or the main risk factors were transformed into categorical or group measures (*n* = 14) are discussed in the next sub-sections.

#### Decisions informing categorisation

Amongst all studies which employed categorisation (*n* = 14), one (7%, CI = 0%, 34%) article explained their choice for reported categories. Categorical groupings adopted in the study were explained as hypothetically driven. Hypothesis-driven categories were then used to construct a cut-off or dichotomised model which was tested against the non-categorical (continuous) model. Otherwise, the rest of the studies, 93% (CI = 66%, 100%) did not explain or state reasons informing their choices of categorisation.

#### Criteria used for categorisation

Criteria used in establishing categorical boundaries for the exposure variables were varied with 21% (CI = 5%, 51%) of the studies using quantiles (e.g., median, quartiles, quintiles, and deciles). Equally spaced intervals or arbitrary groupings (which does not appear to be data or clinically driven) were very popular criterion for deciding categorical boundaries. Both equally spaced interval and arbitrary grouping criterions were observed in 65% (CI = 35%, 87%) of studies were categorisation occurred (see Table 1). Altogether, a combination of articles consisting ordered categories (equally spaced intervals and quantiles) and arbitrary grouping produced 86% (CI = 57%, 98%) of studies.

**Table 1:** Key findings showing the characteristics of categorisation amongst the exposure variables in epidemiological studies

Characteristics of categorisation	% of articles & CI regions
Prevalence of categorisation	61% (CI = 39%, 80%)
Decision informing categorisation	
Hypothesis-driven categories	7% (CI = 0%, 34%)
Unknown (reasons not provided in the articles)	93% (CI = 66%, 100%)
Criteria used for categorisation	
Established external criteria (e.g., WHO standards)	14% (CI = 2%, 43%)
Arbitrary grouping	29% (CI = 8%, 58%)
Equally spaced interval grouping	36% (CI = 13%, 65)
Quantile grouping	21% (CI = 5%, 51%)
Number of categories used amongst grouped exposures	
2	7% (CI = 0%, 34%)
3	7% (CI = 0%, 34%)
4	29% (CI = 8%, 58%)
5	29% (CI = 8%, 58%)
6	14% (CI = 2%, 34%)
10	14% (CI = 2%, 34%)
Proportion of trend testing	57% (CI = 29%, 82%)

Otherwise, the other 14% (CI = 2%, 43%) of articles selected their categories based on established guidelines. For example, Gardner and colleagues [26] used the WHO standards to categorise BMI into four categories; underweight (BMI < 18.5), normal (18.5 ≤ BMI < 25), overweight (25 ≤ BMI <30), and obese (BMI ≥ 30) and Kaukonen and colleagues [27] defined systemic inflammatory response syndrome (SIRS) status (present/absent) based on consensus statement of the American College of Chest Physicians and Society of Critical Care of Medicine.

#### Number of categories

When transforming continuous exposure variables for categorical analyses, the number of categories used across the studies varied between two and ten categories (see Table 1). Studies employing four or five categories were common. For example, Gauffin and colleagues [28] investigated the association between school performance (exposure) and alcohol-related disorders (outcome) in early adulthood population by dividing the population into five categories: high school marks (> mean + 1 SD); high average (between mean and mean + 1 SD); lower average (between mean and mean -1 SD); low (< mean - 1 SD) and missing. The practice of categorisation with four or five categories was found in 57% (CI = 29%, 82%) of the articles. Dichotomisation (or grouping into two categories) was observed in one (7%, CI = 0%, 34%) article whilst ten categories appeared in two (14%, CI = 2%, 43%) articles (see Table 1).

When comparing the practice of categorisation using quantiles against equally spaced interval grouping, four or five categories were more likely to occur with the latter practice. Amongst studies with four or five categories, equally spaced interval grouping occurred in 38% (CI = 9%, 76%) of the articles compared to 25% (CI = 3%, 65%) of quantiles.

#### Trend testing and analysis

Trend tests are often performed to assess the strength of any exposure-outcome relationships that may exist in an investigation [29]. The results show that 57% (CI = 29%, 82%) of the studies which employed categorisation, performed the trend tests. For example, Wang and colleagues [30] performed a trend test in risk estimates using the median values of the heart rate quintile categories. The five values were treated as a continuous measure and were used to evaluate the risk trend; *p*-values were presented as part of the trend testing. In another example, Victora et al. [25] performed the linear trend test based on mean categories for months of breastfeeding.

Amongst all studies were trend testing was performed, various significance trend values ranging between 0.0001 and 0.001 were obtained and interpreted as significant. However, there was variation across studies on how these values were obtained. Guertin and colleagues [31] obtained the overall trend value from the pairwise estimates comparing coffee drinkers (number of cups/day) against non-drinkers (reference group). Moreover, in some studies, floating estimates (where no reference group is assumed) were used to attain the trend values.

#### Covariate adjustment

Considerations were also made to establish the number of confounders or other variables often adjusted for in studies investigating exposure-outcome relationships. Amongst studies where the exposure or main risk factor was categorised, the number of confounders or adjusted variables ranged between 3 and 20 with an average of 10 variables. Cohort or follow-up studies tends to report large numbers of variables or confounders compared to cross-sectional and case-control studies.

### Summary of key findings

Table 1 provides summary statistics of key findings emerging from the study results. The proportions and confidence intervals of main findings explaining the characteristics of categorisation are presented in the table.

## Discussion

The present study indicates a high occurrence of categorisation in epidemiological studies. Amongst the articles investigating the associations between the continuous exposures and disease outcomes, 61% of them transformed the exposure variables into categorical measures for analysis. The results are consistent with those obtained in previous reviews. Pocock et al. [5] and Turner et al. [6] respectively reported 84% and 86% of categorisation in epidemiological studies. However, compared to these studies, we recorded the lowest proportions of categorisation. This could be attributed to the numbers and journals selected for assessment. For instance, the American Journal of Epidemiology (AJE) which was not considered here, contributed more articles (about 53% of articles) in Turner’s study. There is also a possibility of under-representation from other specialist areas since we only used high-ranking journals. High ranking journals may be strict and particular with the quality of work they wish to publish. Thus, this could limit the number of articles considered in our study. However, there are advantages to evaluating high impact journals. They offer us the opportunity to report on practices from leading researchers.

Amongst the transformed continuous exposures, nearly 60% of the articles reported ordered categories (using either equally spaced intervals or quantiles). This kind of categorisation when investigating the exposure-outcome relationship has some disadvantages [14]. Quantiles produce estimates which are data dependent. On the other hand, equally spaced interval groupings produce categories which can be statistically inefficient and unjustifiable. With normally distributed data, it will be ideal to have more categories at the center and few at the tails [14]. One would expect this to be a justification for arbitrary grouping however none was provided for all articles where such criterion was used. Justifications informing categorisation or grouping were explained in 7% of the studies. This is beside the call to describe *why quantitative groupings are chosen in the studies* (recommendation 11 of the STROBE guidelines). Hence, high proportions of articles not explaining their choice for categorisation could be an indication that authors are not aware of existing guidelines. Otherwise, authors are ignoring the guidelines or simply underestimating the consequences of categorising data when analysing continuous variables.

The assessment also shows that researchers use different categories when categorising exposures or risk factors. However, four and five categorical groupings were common amongst studies categorising quantitative exposure variables. Approximately, 60% of the studies used four or five categories when transforming the exposures for analysis. The finding is consistent with what other researchers view as a common practice in epidemiology [19, 32]. According to Royston [19] and Becher [32], four or five categories are often created in the field of epidemiology. Dichotomisation was not popular; the practice featured in one article only.

Of particular interest was also how the confounders and other variables were adjusted when investigating the exposure-outcome relationships. There are no clear procedures to decide on the choice and number of confounders and other variables when investigating exposures and outcome relationships [33]. Quite often we rely on evidence from other studies, subject knowledge, statistical packages and correlations to choose the variables we wish to include as confounders in our analysis. In this study, we observed large numbers of unrelated confounders and variables being investigated. This could result in false positive claims. Careful consideration is needed to establish what true confounders are in our investigations. In one article in this assessment [31], we observed a multivariable model being adjusted for 20 variables. Such models are hard to interpret and can be misleading. Variables might be dependent on each other making it difficult to explain their associations. The use of directed acrylic graphics (or DAGs) [34] offers a better solution to identify and establish relations. DAGs provides graphical models explaining causal relationships amongst variables of interest [34]. Furthermore, studies with a large number of confounders and variables should also be accompanied by large samples. The samples should also incorporate the study designs. Otherwise, studies with small samples, categorising exposures and having too many variables are likely to be underpowered [19].

Taking into consideration trend testing and analysis, 57% of the articles performed the tests after categorising the exposure variables. Trend values such as ordinal scores, mean and median of categories were often used in fitting and evaluating the overall trends. In all the studies reviewed, the null hypothesis was not clearly provided. However, indications from the studies suggest the hypothesis of no exposure-disease association was always assumed. We found that small significance values for trend statistics were in some studies interpreted as the existence of a monotonic (continuously increasing or decreasing) relationship between the exposures and risk outcomes. For example, after obtaining a trend value of 0.0006, Liu and colleagues [35] concluded that the risk between nasopharyngeal carcinoma (NPC) and categorised sibling size was continuously increasing. Such interpretations could be misleading. Sometimes a significant trend statistic value does not imply a continuously increasing risk of exposure on the outcome. Trend tests are not tests for monotonic exposure-outcome relationships [36, 37]. If the exposure-outcome relationship is unknown, the trend test may obscure rather than reveal the relationship [36]. Trend or slope estimation methods such as polynomial regression and non-parametric models should supplement trend testing when investigating relationships which are unknown.

## Conclusions

In epidemiology, studies evaluating issues of categorisation according to the STROBE guideline are lacking. Based on recommendation 11 of the STOBE guidelines, our study highlights current practices for analysing quantitative variables focusing on issues of categorisation. Findings obtained using five medical journals indicates high proportions of categorisation within epidemiological studies. Categorisation of continuous exposure or risk factors was found in 61% of articles assessed. Reasons and justifications informing the choices and practices of categorisation are rarely provided and remain unknown. The findings confirm the presence and claims of categorisation viewed by some researchers as a dominant feature for analysing continuous data in medicine.

Clearly, these findings raise concerns about the adequacies of analysis and quality of reporting. Categorisation enables researchers to assume simple relationships between the outcome and exposures and in the process the information is lost. How much information is lost will depend on cut points or categories used [38]. In our study, we have seen four or five group categories being dominant. However, we cannot be certain on how much of the information is lost when four or five group categories are assumed under different exposure - outcome associations.

The majority of researchers also preferred to use equally spaced intervals or arbitrary grouping. In medicine, biologically meaningful cut points are necessary to inform decisions which relate to the pattern of the data. Establishing meaningful cut points where complex relationships or associations are present may not be easy. Alternative approaches such as fractional polynomials [39, 40] and splines [41, 42] are available. However, the precision and performance of these approaches in the presence of complex associations are also not well known [43]. Further research evaluating these approaches, their performance and precision under different complex associations is required.

Other existing guidelines available for medical researchers can be found on online resources including the Enhancing the QUAlity and Transparency Of health Research (EQUATOR) network website (www.equator-network.org) which have the aim of improving the reporting of epidemiological and clinical studies.

## Additional file

Additional file 1: A standard from used in the study for data collection. (DOCX 74 kb)
