# Erratum to: Assessing the reporting of categorised quantitative variables in observational epidemiological studies

**DOI:** 10.1186/s12913-017-2237-9

**Published:** 2017-04-26

**Authors:** Onkabetse V. Mabikwa, Darren C. Greenwood, Paul D. Baxter, Sarah J. Fleming

**Affiliations:** 1 Division of Epidemiology and Biostatistics, LICAMM, School of Medicine, University of Leeds, Leeds, UK; 2 Section of Epidemiology and Biostatistics, LICAP, School of Medicine, University of Leeds, Leeds, UK

## Erratum

Upon publication of the original article [1], it was noticed that on page 3 in Fig. 1, the textbox appearing with the word “methodological” should read “Identification”. This has now been acknowledged and corrected in this erratum. This has now been incorporated in the new Fig. 1 shown below.

**Fig. 1:**
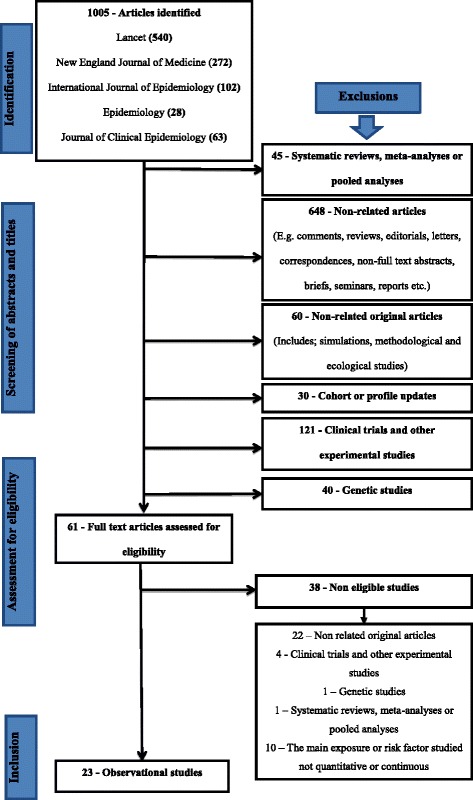
A detailed flow chart summarising the selection and identification process of eligible articles
