# Supplementary material for: Assessing the reporting of categorised quantitative variables in observational epidemiological studies
Source: BMC Health Serv Res. 2017 Mar 14;17:201. doi: 10.1186/s12913-017-2137-z (PMC5348776; doi:10.1186/s12913-017-2137-z)
Supplement: Additional file 1: — A standard from used in the study for data collection. (DOCX 74 kb) [file 12913_2017_2137_MOESM1_ESM.docx]

**Data collection form**

**Section 1: General information**

D.O.I (Digital Object Identifier) Reviewers

1: O.V.M

2: S.L.F

3: P.D.B

4: D.C.G

(4) Does this article satisfy the eligibility criterion?

1: Yes

2: No

***(If Yes, skip to Q6 otherwise stop entry at Q5)***

(5) If No, which eligibility criterion are not satisfied? (5a) If other, specify

1: Sample size too small

2: Analysis based on pooled data/estimates (e.g. systematic reviews & meta-analysis)

3: Main investigation based on effect modification or interaction effects

4: Based on experimental or randomised control trial designs

5: Non-relevant article (e.g. comments, reviews, correspondences, tutorials, seminars etc.)

6: Non-full text abstract (e.g. poster/conference/meeting abstracts)

7: Cohort or profile update studies

8: Article not relevant

9: Other

(6) Journal

1: International Journal of Epidemiology

2: Epidemiology

3: Journal of Clinical Epidemiology

4: New England Journal of Medicine

5: Lancet

(7) Types of study design (7a) If other, specify

1: Cohort

2: Case-control

3: Cross-sectional (8) Sample size (no.of participants)

4: Other

**Section 2: Outcomes**

(9) The principal disease outcome or condition studied

(10) Type of outcome (10a) If other, specify

1: Binary: absent/present event (yes/no)

2: Time-to-event or events/person-year

3: Ordered categorical

4: Unordered categories

5: Continuous or quantitative measure

6: Other

(10b) For continuous outcomes, how were they analysed?

1: Quantitatively/Continuously

2: Qualitatively/Categorical/Grouping

3: Both

4: N/A

**Section 3: Exposures**

(11) Name of the main exposure or risk factor studied

(12) Nature of the main exposure or risk factor studied (12a) If other, specify

1: Lifestyle

2: Environmental

3: Pre-existing condition

4: Diet

5: Biochemical

6: Physiological

7: Socioeconomic

8: Genetic

9: Other

(13) Type of main exposure or risk factor studied (13a) If other, specify

1: Ordered categorical

2: Unordered categories

3: Continuous or quantitative

4: Other

(14) If the main exposure or risk factor is continuous and was categorized, how many groups were used?

***Skip to Q22, if exposures are categorical - (for both unordered & ordered categories)***

**Details of continuous or quantitative exposure/risk factors**

(15) Is the main exposure/risk factor truly quantitative?

1: Yes

2: No

(16) Was it measured or collected quantitatively?

1: Yes

2: No

9: Not specified/Unknown

(17) How was the main exposure/risk factor analysed? (17a) If other, specify

1: Quantitatively or Continuously

2: Categorically or Ordered categories

3: Both

4: Other

**If analysed categorically;**

(18) What informed this approach or decision? (18a) If other, specify

1: Data driven or evidence based categories

2: Hypothesis driven categories

3: N/A

4: Other

9: Not specified

(19) How are the categories analysed, are they ordered categories? (19a) If No, specify

1: Yes

2: No

3:N/A

(19b) Have they looked at the trend?

1: Yes

2: No

3:N/A

9: Not specified/Unknown

(20) Specify the types of grouping that were used or assumed? (20a) If other, specify

1: Statistical criteria (e.g. equal sized group)

2. External basis (well established e.g. WHO criteria)

3. Logical grouping/Equally spaced intervals (e.g. 10 year age groups)

4. Random/Arbitrary grouping

5. Quantiles

6: Other

**If analysed quantitatively;**

(21) Was linearity or nonlinearity between the continuous exposure and the outcome considered?

1: Yes

2: No

3:N/A

9: Not specified

**Section 4: Details of the analysis**

(22) Specify if any, the model used for producing the estimates between the exposure and the outcome

(23) What were the principal types of statistical results used for reporting the main exposure/risk factor?

1: OR (binary or per unit change in risk factor)

2: RR

3: Difference in % or rates

4: Hazard ratios or other time-to-event measures or rate ratios (e.g. from Poison)

5. Regression: difference in means or regression coefficients

6: Other

(23a) If Other, specify

**Adjustment for other variables/confounders**

(24) Are there any other variables considered in the study?

1. Yes

2. No

(25) If Yes, how many are they?

(26) Are there any continuous risk factors analysed as categorical variables?

1. Yes

2. No

(27) If Yes, how many continuous risk factors are analysed as categorical variables?

**Presentation of results**

(28) How are the results using the categorized principal risk factor or exposure presented?

1: Tables

2: Figures

3: Both

4: Other

(28a) If other, specify

**Types of estimates**

1. (b). (c).Reference group

1: Yes 1: Yes 1: Lowest

2: No 2: No 2: Middle

3: SE's 3: Highest

4: Other

9: N/A

(29) Continuous e.g. regression coefficients

(30) By group for all groups

(31) By group relative to reference group

(32) Other, specify

**P-values and other statistical significance tests**

**Types of tests**

1: Yes

2: No

3: With CI

(33a) None

(33b) Continuous analysis p-value

(33c) Trend test (i.e. scores for groups)

(33d) Pairwise p-values (i.e. every group relative to reference group)

(33e) Global p-values (e.g. LRT model with all dummy vs w/t all dummy)

(33f) Other, specify

(34) Any other comments?
